# Evaluation of a Computer-Tailored Healthy Ageing Intervention to Promote Physical Activity among Single Older Adults with a Chronic Disease

**DOI:** 10.3390/ijerph15020346

**Published:** 2018-02-15

**Authors:** Janet M. Boekhout, Brenda A. J. Berendsen, Denise A. Peels, Catherine A. W. Bolman, Lilian Lechner

**Affiliations:** Faculty of Psychology and Educational Sciences, Open University of the Netherlands, Heerlen, PO Box 2960, Heerlen 6401 DL, The Netherlands; brenda.berendsen@ou.nl (B.A.J.B.); denise.peels@ou.nl (D.A.P.); catherine.bolman@ou.nl (C.A.W.B.); lilian.lechner@ou.nl (L.L.)

**Keywords:** healthy ageing, intervention, computer-tailoring, physical activity, older adults, single, chronic disease, physical impairment

## Abstract

This study explores the effectiveness of the Active Plus65 intervention designed to stimulate physical activity among single older adults with a chronic physical impairment. A quasi-experimental pre-test post-test study was performed. The intervention group (*n* = 411; mean age = 76.75; SD = 7.75) was assessed at baseline, three months, and six months. Data of comparable older adults who completed the original Active Plus intervention served as reference group (*n* = 87; mean age = 74.36; SD = 6.26). Multilevel regression analyses were applied: outcome measures were weekly minutes of moderate to vigorous physical activity (MVPA) and days per week with at least 30 min of MVPA. Although Active Plus65 did not outperform the original intervention, in itself Active Plus65 effectuated a significant increase in the weekly minutes of MVPA (B = 208.26; *p* < 0.001; Effect Size (ES) = 0.45) and in the days per week with sufficient MVPA (B = 1.20; *p* < 0.001; ES = 0.61) after three months. After six months, it effectuated a significant increase in the days per week with sufficient MVPA (B = 0.67; *p* = 0.001; ES = 0.34) but not for the weekly minutes of MVPA (*p* = 0.745). As Active Plus65 increased MVPA at three months with a higher ES than average interventions for this vulnerable target group, it potentially makes an interesting intervention. Further development should focus on long-term maintenance of effects.

## 1. Introduction

It is expected that by 2040 the number of people older than 65 will have doubled worldwide [1]. Aging populations come with high costs for society, as health care expenditure rises with age, showing a steep incline from the age of 65 [2]. One of the reasons of this increase in health care expenditure is that the majority of people develop one or more non-communicable chronic disease(s) (NCCD) later in life [3,4]. Literature shows no uniform definition of what a NCCD is. A frequently used definition states that “chronic diseases are generally characterized by uncertain etiology, multiple risk factors, a long latency period, a prolonged course of illness, noncontagious origin, functional impairment or disability, and incurability” [5], which, similar to most definitions, includes the presence of some degree of functional impairment regarding physical activity (PA) [6]. Being sufficiently physically active can prevent or postpone the development of several NCCDs and has a beneficial effect on the course of already present NCCDs [7,8,9,10]. For older adults (65 years and over), sufficient PA has additional beneficial effects. The risk of developing cognitive decline is lower and balance is preserved longer, reducing the risk of fall-related injuries [11,12]. However, the number of adults that achieve the recommended level of PA (defined by the World Health Organisation [8] as a minimum of 150 min per week of moderate to vigorous physical activity (MVPA), which is comparable to the Dutch recommendation [13] of a minimum of 30 min of MVPA per day on at least five days per week), decreases steadily as people age [3,14]. This is especially the case for older adults with a NCCD, who are often confronted with physical impairments and are thus facing additional barriers regarding PA [15,16,17]. In the Netherlands, 84% of healthy people over the age of 55 achieve the recommended level of PA; for people with a NCCD, this is 71%, and for people with a physical limitation caused by a NCCD, this is 42% [18]: Western society in general shows similar figures [19]. Besides older people who have a NCCD, older people who are single are also reported to be less physically active than those living with a partner [20,21,22]. 

The subpopulation of single older adults with a NCCD therefore deserves special attention regarding their health: not only is this group highly vulnerable for health problems associated with a lack of PA, but also because of its present and future proportion in society. Although much research is available on health promotion interventions, and healthy ageing has a strong scientific and societal relevance, the specific strongly growing target population of single older adults with a chronic limitation seems to have been often overlooked in research. Promoting PA by means of eHealth interventions is a relatively low-cost way to improve the health of this subpopulation. Although research is available regarding the effectiveness of eHealth interventions for older adults, so far research has mainly focused on the younger age groups of older adults (i.e., 50–70 years) or on multiple health behaviors instead of only PA [23]. Also, research often focusses on health behaviors with a particular disease [24], whereas multi-morbidity is highly prevalent [25]. The current study provides insight in the effectiveness of the Active Plus65 eHealth intervention, which is an adapted version of the proven effective Active Plus50 intervention. The intervention is adapted to better suit the needs of single people aged over 65 with a chronic disease [26] by, among other adaptations, stimulating participants to be physically active together with others in order to prevent or decrease loneliness, which is an independent risk for mental and physical health. To our knowledge, no interventions with a focus on this specific highly vulnerable and growing target population have been researched so far. 

The present study provides insight into the effects of the Active Plus65 intervention on PA and it examines whether the adaptations made to Active Plus65 result in comparable or even better results than the original proven effective intervention that was less adapted to this specific target population. Although a large amount of literature is available on health promotion interventions that were proven successful in randomized clinical trial (RCT)-settings, rather limited research is available regarding the effectiveness of the same interventions in real-life implementation settings. As Active Plus50 has been extensively studied (and proven effective) in a research setting [27,28], the current study is performed in a real-life setting to provide a realistic insight into the potential effects of the Active Plus intervention on public health [29,30]. 

## 2. Materials and Methods

### 2.1. Intervention

Active Plus65 is a computer-tailored healthy aging intervention designed to stimulate or maintain sufficient PA among single older people with an impairment in PA caused by a NCCD. The tailored advice can be delivered either in an internet-based version or in a printed version and is presented in a predominantly text-based format, supplemented with graphs, pictures (printed version), or short videos (internet-based version). There are no differences between the content of the printed or internet-based version. The tailored information is based on the participants’ demographic and psychosocial characteristics (e.g., age, attitude, motivation, and self-efficacy regarding PA), their present PA level, and the stage of behavioral change they are in [26]. Depending on the assessed characteristics and determinants, the dispensed form of each advice consists of 7 to 12 pages (A4-format). In addition, each advice comes with activating elements such as (1) planning sheets that the participant is stimulated to use in order to plan PA; (2) formats that the participant has to fill out in advance on how they plan to deal with difficult situations that may interfere with PA; (3) formats where they are asked to formulate and write down their implementation intentions; (4) brochures from local PA-exercise groups; and (5) medical information on exercising with a physical limitation.

During the intervention period of four months, participants receive personal PA-advice on three occasions, based on two assessments using self-report questionnaires. The first advice aims to raise consciousness of the current level of PA by targeting pre-motivational psychosocial constructs, such as awareness and knowledge. The second advice motivates participants to increase physical activity by targeting motivational psychosocial constructs such as attitude, self-efficacy, social influence, intrinsic motivation, and intention. Moreover, participants are stimulated to plan their PA, and to prepare for difficult situations. The intervention also helps to overcome barriers with regard to PA, and thus helps the participants to transfer their motivation into sustainable behavior: depending on the degree in which participants are already active, this is done at the second or the third advice stage by targeting post-motivational psychosocial constructs, such as strategic, action-, and coping-planning. A follow-up assessment is performed six months after the start of the intervention: this assessment serves only to measure the level of PA and does not result in the provision of advice. 

Active Plus65 was developed in 2016 [26] using the Intervention Mapping Protocol [31], and is rooted in influential health behavior change theories, such as the I-Change model [32], transtheoretical model [33], self-determination theory [34], self-regulation theory [35], and health action approach [36]. Active Plus65 is an adaptation of the Active Plus50 intervention, which was previously developed to stimulate the initiation and maintenance of PA for people over the age of 50 [37,38]. Although Active Plus50 was proven effective for the general population of people over the age of 50 [27,28], showing higher effect sizes (ESs) for people aged over 65 than younger age groups, program evaluations showed that single older adults with an impairment in PA caused by a NCCD preferred more information about the possibilities for PA that match their impairment and about increasing their social network while being physically active [27,28]. These preferences are in line with other research that has shown that effectiveness of eHealth interventions for older adults increases with the level of individual tailoring and if the advice contains a referral to local possibilities for PA [39]. Based on these findings, Active Plus65 tailors more in-depth to the physical impairments that the participants report, motivates participants to find other persons to be physically active with, and refers to local activities where one can be physically active, preferably in a social group. 

### 2.2. Study Design and Procedures

As Active Plus50 has been proven effective in a RCT [27,28], the study of Active Plus65 was conducted in a real-life implementation setting. The restrictive setting and standardized protocols of RCTs can result in an over- or underestimation of the effectiveness of interventions when compared to studies that are performed in an implementation setting. This is due to several differences in the characteristics of RCTs and implementation settings [40,41,42]. One of these differences is that the motivations of participants who are aware that they are part of a scientific study may differ from the motivations of participants that join an intervention in real life. Also, intervention and control groups often undergo an intensive screening to determine eligibility and may thus have different features than people in a real-life setting. The current design thus provides a realistic insight into the effects that Active Plus65 may have on public health [29].

A quasi-experimental pre-test post-test study was conducted with three assessment time points. The assessment time points during the intervention-period were at baseline (T0), at three months after baseline (T1), and a follow-up assessment at six months after baseline (T2). The study of Active Plus65 was conducted in a real-life implementation setting and compared to existing data from a previously conducted RCT of Active Plus50. 

Participants for the intervention group were gathered from a Dutch municipality. All inhabitants who, according to the municipal data, were single and 65 years or older (*n* = 6751) were invited in April 2016 by direct mailing by their municipality to participate in Active Plus65. Invitations were sent by post, containing a personalized information letter including log-in details for internet-based participation and a prepaid response card to request a paper questionnaire for those preferring the printed version of the intervention. Inclusion of participants lasted from early April until the end of May 2016. Advice was sent to the participants immediately after completing the baseline assessment for the internet-based version (in their web browser and by email) or within two weeks after returning the completed printed questionnaire (by post). After three months, all participants received the second questionnaire: this was sent by the same method the participants had used for the first questionnaire (i.e., email or printed mail). The time schedule of sending the advice was similar for sending the advice for T0. Six months after baseline (i.e., two months after the end of the intervention-period), all participants received a follow-up questionnaire. 

As Active Plus65 was designed to better meet the wishes and needs of the specific target population [26] in comparison to its predecessor, Active Plus50 (which was designed for all adults aged over 50, regardless of their marital status or the presence of a NCCD), Active Plus65 was compared to Active Plus50. For this comparison, the Active Plus50 data from a previously performed randomized clinical trial (RCT) [28] was used. From this data, a reference group was created by extracting only the data of those participants of the intervention group of Active Plus50 who were aged over 65 years, single, and living with a physical impairment caused by a NCCD. Due to the implementation of the Active Plus65 in real-life, no control group was available, and the number of participants in the control group of the original Active Plus50 studies that met the requirements was too low to supply sufficient power in the analyses. In line with the recommendations of Curran et al. [29] and Glasgow et al. [30], this study compares an adapted version of the intervention in a real-life implementation setting with its predecessor that has been proven effective in an RCT. Such a blending of study design components has been proposed previously: Curran et al. [29] describe the benefits that “effectiveness-implementation hybrid designs” have. These hybrid forms could prevent the possibility that interventions that are proven effective in the controlled setting of an RCT do not properly address the issues that determine its effectiveness in implementation settings. The current study design meets the description and assumptions of such a hybrid design (i.e., examining the effects of an intervention in an implementation setting while taking into account the outcomes gathered in a RCT setting). 

**Figure 1:**
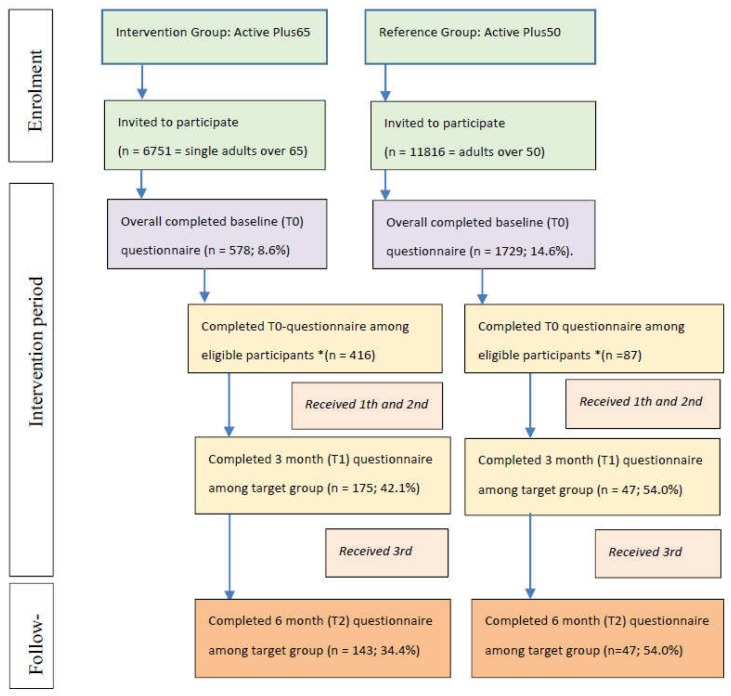
Flow chart of reach and attrition in intervention and reference group (Note: T0 is reported as percentage of invited participants; T1 and T2 are reported as percentage of baseline numbers; * Eligible participants are those that meet all requirements of being single, over the age of 65, and chronically impaired in PA).

There were some differences in data collection for Active Plus65 and Active Plus50. In the invitation letter of Active Plus65, it was explained that the intervention was specifically designed for single, chronically impaired people aged over 65, whereas in Active Plus50, the general population of people aged over 50 was targeted. Contrary to Active Plus65, where participants could choose themselves between a printed or internet-based program, the participants in Active Plus50 had been randomized to either a printed or internet-based intervention group or a control group. Despite these differences, Active Plus50 can be considered a fitting reference group. Figure 1 presents an overview of the reach and attrition of intervention and reference group. From both data sets, only eligible participants (i.e., participants who met the criteria of being single, over 65 years, and chronically impaired) were analyzed.

A sample size calculation [43] showed that inclusion of a total of 296 participants for the between groups comparison was required (ES = 0.29, power = 90%) for the outcome-measure of weekly minutes of MVPA. The ES and power are based on the previous study into the effectiveness of Active Plus50, where an average ES of 0.29 was found on this outcome measure [28]. For weekly days with sufficient PA, the average ES was 0.22, resulting in a required total of 514 participants. Based on the same parameters, 130 participants were required for the within group analyses.

All subjects gave their informed consent for inclusion before they participated in the study. The study was conducted in accordance with the Declaration of Helsinki, and the protocol was approved by the Research Ethics Committee of the Open University of the Netherlands (reference number U2016/02373/HVM). The data from Active Plus50 received approval under the Dutch law for medical scientific research (reference number NTR2297) [37].

### 2.3. Measures

#### 2.3.1. Outcome Measures

Outcome measures are the weekly minutes of MVPA and the days per week with sufficient MVPA, both measured using the Short Questionnaire to Assess Health Enhancing Physical Activity (SQUASH) [44,45,46]: The relative validity (*rSpearman* = 0.45; 95% CI = 0.17–0.66) and reproducibility (*rSpearman* = 0.58; 95% CI = 0.36–0.74) of this questionnaire can be considered to be reasonable. The SQUASH was filled in based on an average normal week in the last month. PA was scored on four types of activities, i.e., during commuting by foot and bicycle, at (volunteering) work or study, during domestic chores, and during leisure-time/sport activities. The participants reported on how many days a week they performed this activity (in a number between 1 and 7), how much time per day this took (in hours and/or minutes), and how demanding these activities were (with three options; *light*, *moderate*, and *vigorous).* The outcome measure of weekly minutes of MVPA is calculated by multiplying the frequency (days per week) and duration (hours/minutes per day) of activities that were performed with moderate to vigorous intensity. The outcome measure of days per week with sufficient MVPA was measured by a single item in the questionnaire: “how many days per week (on average in the past month) are you, in total, at least moderately physically active by undertaking, for example, brisk walking, cycling, household chores, gardening, sports or other physical activities for at least 30 min?”.

#### 2.3.2. Demographics

Age, gender, educational level, Body Mass Index (BMI), intention to be sufficiently physically active, way of entry (internet-based or printed), degree of impairment, and the two outcome-measures were assessed at baseline. Educational level was categorized into low (elementary, medium general, preparatory vocational, lower vocational, higher general secondary, preparatory academic education, medium vocational school) and high (higher vocational school or university) according to the Dutch educational system. BMI is the division of self-reported weight by height in meters squared. The intention to be sufficiently physically active was measured by three items on a 10-point scale, ranging from 1 (*absolutely not*) to 10 (*absolutely sure*); an example of such an item is: “How likely do you think it is that you will stay or become sufficiently physically active?” [47,48,49,50]. The degree of impairment was measured in different ways in Active Plus65 and Active Plus50. In Active Plus65, the participant was asked for 10 prevalent NCCDs to state to what degree he/she is limited in his/her physical activity by one of the mentioned diseases or by another not mentioned disease. For each disease, the participant could score the degree of impairment on a 4-point scale ranging from 1 = not at all/hardly, 2 = a little, 3 = very, to 4 = extremely: the highest reported level of impairment on the stated NCCDs determined the degree of impairment. Though the above is not a validated question, its usability has been successfully pilot tested among the target population [26]. In Active Plus50, the degree of impairment of all potentially present NCCDs was measured by one single item (“To what degree are you impaired in PA?”) on a comparable 4-point scale as in Active Plus65.

Furthermore, all psychosocial variables necessary to provide the participants with tailored advice (e.g., one’s attitude, motivation, and self-efficacy regarding PA) were assessed, but will not be elaborated on, as these are not included in the analyses of the current study.

### 2.4. Statistical Analyses

All analyses were conducted in SPSS for Windows (version 22) (IBM Statistical Package for Social Sciences, Armonk, NY, US). In all tests, a reproducibility level of 95% was applied (α = 0.05). Analyses were applied without imputation of missing data, as applying multilevel analyses to an incomplete dataset has been shown to result in more accurate estimations than using multiple imputation [51]. Baseline differences (on days per week with sufficient MVPA, weekly minutes of MVPA, age, gender, education, BMI, way of entry, impairment, and intention) between participants of Active Plus50 and Active Plus65 were analyzed by t-tests and Chi-square tests. Binary logistic regression was applied to test for selective drop-out for the same variables as the analyses for baseline differences. As measurement points were nested within participants, resulting in possible interdependence, multilevel linear regression analyses were conducted with random intercepts (time and participants) to study the intervention effect on PA (within group comparison with two separate analyses, i.e., one per dependent variable), and to compare the differences between the intervention group and reference group (between group comparison with two separate analyses, i.e., one per dependent variable). Dependent variables were days per week with sufficient MVPA and weekly minutes of MVPA. Intervention effects were compared for differences between T0 and T1, and between T0 and T2. In the analyses of the between groups comparison, the independent variables were the dummies of the different groups (Active Plus65 and reference group), baseline value of PA, and the a priori selected covariates (gender, educational level, BMI, intention, and way of entry (internet-based or printed)). In the within group analyses, the same independent variables were applied, with exclusion of the dummy for the different groups. Cohen’s *d* ESs were calculated, in which ESs were defined as the mean differences in PA between T0 and the following measurement (i.e., T1 or T2) divided by the pooled standard deviation (SD) of those means [52]. For the ESs of change in PA within each group (Active Plus65 and reference group), T1 and T2 were compared to T0 with an ES calculator for within group effects [53]; for the between groups comparison of the ESs of the intervention group with the reference group, their respective individual means and SDs were used in an ES calculator for between groups effects [54]. ESs of 0.20, 0.50, and 0.80 were considered to be, respectively, small, medium, or large [55].

## 3. Results

### 3.1. Characteristics of the Study Population

An overview of the flow of participants in this study is presented in Figure 1. The way of entry was the only significant predictor of drop-out, with online participants less likely to fill in the 6 month questionnaire (B = 0.663; *p* = 0.003). At baseline, participants in Active Plus65 were older, more often male, more often perceived severe physical impairments, and had fewer days per week with sufficient MVPA compared to the reference group (Table 1). 

### 3.2. Intervention Effects on PA

The within group analyses revealed that overall, the days per week with sufficient MVPA of the Active Plus65 group increased significantly at T1 compared to T0 (B = 1.20; *p* < 0.001; ES = 0.61). Weekly minutes of MVPA also increased (B = 208.26; *p* < 0.001; ES = 0.45). Between T1 and T2, the days per week with sufficient MVPA decreased, but the total increase between T0 and T2 was still significant (B = 0.67; *p* = 0.001; ES = 0.34). For weekly minutes of PA, the decrease between T1 and T2 was larger, causing the overall effect between T0 and T2 to become non-significant (B = −17.3; *p* = 0.745). ESs are small, except for the ES for days per week with sufficient MVPA at T1, which has a medium ES. 

In the between groups analyses, no significant differences between Active Plus65 and the reference group were found for the days per week with sufficient MVPA between T0, T1, and T2 (Table 2). For the weekly minutes of MVPA, a significant difference between Active Plus65 and the reference group was only found between T0 and T2 (B = 370.94; *p* = 0.004; ES = 0.25), in favor of the reference group: the ES can be considered small. 

For all analyses, age and intention were significant covariates: a higher increase in days per week with sufficient MVPA and in weekly minutes of MVPA was seen when participants were younger and had a higher intention to be physically active. For days per week with sufficient MVPA, BMI was also a significant covariate: a higher increase in days per week with sufficient MVPA was seen when participants had a lower BMI. Gender was an additional significant covariate for weekly minutes of MVPA, where a higher increase was seen for males.

## 4. Discussion

The present study evaluated the potential of the Active Plus65 intervention to affect PA. Active Plus65 was compared to the proven effective Active Plus50 intervention in order to determine whether the effects of the adaptations that were done to Active Plus50 to better meet the needs of the specific target population [26] showed results. This study provides insights into the feasibility of interventions that are specifically designed for the vulnerable, growing, and so far often overlooked target group of single older adults with a chronic impairment in PA.

### 4.1. Intervention Effects on PA

The Active Plus65 intervention group in itself showed a significant increase in PA for the days per week with sufficient MVPA at three months as well as at the follow-up measurement at six months in comparison to the baseline. Although weekly minutes of MVPA also showed a significant increase after three months, at six months this effect had almost completely evaporated. This discrepancy between the maintenance of the increase of days with sufficient PA but decrease in weekly minutes of MVPA after six months may indicate that in the course of the intervention, participants did not increase their total amount of PA but distributed their PA more evenly over the week. A more evenly distributed amount of PA is in line with recommendations to decrease sedentary behavior [56], which is increasingly emerging as an independent risk factor to health [57,58]. Furthermore, the comparison of single bouts of exercise with energy-expenditure matched PA activities that are distributed more evenly over a time-period has been reported to result in more favorable health outcomes for the latter [59]. The outcome measure of weekly days with sufficient MVPA, entailing a more evenly distributed amount of PA, therefore seems relatively more relevant than the outcome measure of weekly minutes of MVPA. In Active Plus65, the outcome measure of weekly days with sufficient MVPA is measured with a single-item question: research supports the validity and reliability of single-item self-reports of PA [60,61], as they often result in a more accurate measurement of PA than multiple items that determine the total weekly minutes of MVPA. The finding that weekly days with sufficient PA did show an increase in Active Plus65 might therefore be more valuable in determining the effectiveness of Active Plus65 than the finding that the increase of weekly minutes of MVPA had almost evaporated after six months. The decrease in PA in the within groups analyses between three months and six months in this study is in line with other interventions aimed at PA, where generally effectiveness on the longer term decreases [62]. Although the long-term sustainability of lifestyle interventions is of major importance to achieve a sustained impact on public health, and several behavior change models have shown that post-motivational factors might play an important role in the maintenance of behaviors [63], research was not able to identify a single factor that determines long-term sustainability of health behavior change [64]. The study of Brouwer et al. [65] demonstrates, however, that, in interventions for older populations, personal contact in the form of follow-up phone calls shows promising effects on the long-term sustainability of intervention effects. This was also the case for personal contact in the form of email interaction with intervention supervisors [66,67,68]. As including personal contact would come with higher costs, providing additional automated (e) mailed advice between T1 and T2, or even thereafter, may potentially be a way to increase the long-term effectiveness of Active Plus65 without adding substantial implementation costs to the intervention: previous studies [66,67,68] have shown promising results regarding the sustainability of intervention effectiveness when automated emails are added.

The ES of Active Plus65 in itself on days per week with sufficient MVPA after three months is 0.61. This is high when compared to the findings in the review of Chase [69], where an average effect of 0.23 was found for single group pre-post intervention studies for older adults. A possible explanation for the higher ES of Active Plus65 may lie in the additional tailoring that has been made to enable PA with a physical impairment. The target group may be in greater need for fitting interventions than the general population of older adults: the relatively high ES may show that Active Plus65 is especially suited for this target group. It may also be an indication that this group is fairly motivated, which is underwritten by the positive baseline assessment of intention, especially when presented with an intervention that fits their needs and requirements. 

Although we were not able to compare the effect of the Active Plus intervention to a no-intervention control group, the ES found in the current study is also higher than the ES of a previous study on Active Plus in which a control group was included [28]. 

Although the Active Plus65 group in itself showed a relatively high increase in PA, it did not achieve better results than observed in the reference group of its proven effective predecessor, Active Plus50. When comparing both interventions, there were no significant differences in effectiveness over time, except on the weekly minutes of PA at the six months assessment, where Active Plus50 outperformed Active Plus65. The finding that Active Plus65 did not show a higher increase in PA than Active Plus50, despite the efforts that were made to better suit this target population when designing Active Plus65 [26], may be explained by the baseline differences and the way participants were recruited, and, consequently, by the representativeness and characteristics of the participants included in the different groups. The recruitment for Active Plus65 emphasized that the intervention was only meant for people with a chronic impairment in PA. Although for the current study only those participants who were over the age of 65 years as well as single and physically impaired by a NCCD were extracted from the intervention group of Active Plus50, the recruitment of Active Plus50 targeted everyone over 50 years of age, impaired or not. Possibly for Active Plus65, this appealed to participants with a higher degree of impairment, as they may have been especially drawn to this intervention. Baseline analyses confirm that 52% of the Active Plus65 participants were very to extremely impaired in their PA behavior caused by a NCCD; in Active Plus50, this was 33%. Participants in Active Plus65 also had a significant lower amount of days per week with sufficient PA at baseline, which may also be an indication of a higher degree of impairment. A higher degree of impairment could result in a lower potential to increase PA. Conn et al. [70] showed that the effectiveness of PA interventions among older adults with a NCCD varies substantially between different groups of chronic diseases. While Conn et al. [70] do not examine the degree of impairment caused by NCCDs, there may be a link: a mild case of one NCCD may be more physically disabling than a severe case of another NCCD. Participants in Active Plus65 were older also than the participants in the reference group: this could also account for a difference in PA, as effectiveness of PA interventions is found to be lower in older age groups [71]. Participants in Active Plus65 may thus have had a lower overall potential to increase PA when compared to the participants in Active Plus50. As there were differences in the way of measuring the degree of impairment, this could not be incorporated into the analyses. In future research on interventions for older adults with impairments, more in-depth analyses on the effects of impairments on PA is called for: at present there is no validated, or even widely accepted, instrument to measure the degree of impairment caused by NCCDs [6], making comparisons with other research difficult. 

### 4.2. Methodological Issues

As far as could be determined by the researchers, this is the first study that evaluates a computer-tailored healthy aging intervention aiming to stimulate PA for single older adults with a chronic impairment in PA. This group will become a major target population for public health enhancing interventions, as the average age in Western societies is increasing rapidly, and thus also the amount of people with one or more NCCDs and impairments caused by NCCDs. To our knowledge, research so far has not filled the need for healthy ageing interventions for this vulnerable group, as studies so far have predominantly focused on younger age groups of the older adults or multiple health behaviors [23], or on people with a specific chronic diseases [24], whereas multi-morbidity is highly prevalent [25]. Furthermore, PA was measured on two outcomes by using a validated instrument. 

Despite these strengths, the study design has some limitations. First of all, the robustness of our study design is not as high as a RCT. However, by adding a reference group we have attempted to address the most important methodological issue of pre-test post-test designs, i.e., not being able to compare to a control group. Moreover, it has been established that real-life implementation settings, such as in our study, overcome disadvantages associated with RCTs, such as not addressing issues that the intervention may encounter in a community setting [30,40,41,42]. Our study is in line with recommendations to apply a hybrid design, where an intervention is evaluated in a real-life setting after effectiveness has been established in a RCT [29]. The difference in outcomes could thus partially be explained by the different research settings, where Active Plus50 was tested in the setting of a RCT, and Active Plus65 was tested in a community setting: as described by Glasgow et al. [30], this could account for either an over- or underestimation of effects. 

Secondly, the study showed a considerable degree of attrition. However, this is common in eHealth interventions, and comparable to other studies with a similar design [72]. By conducting multilevel analyses in this study, the most accurate way of handling missing data was applied [51]. Thirdly, self-report-questionnaires are prone to bias [65,73]. However, for monitoring PA, self-reported data are pragmatic and generally considered appropriate when studying large populations [74]. Moreover, the use of more objective measurement instruments, such as accelerometers, is usually more stressful, especially for older populations [75]. Another advantage of self-reported questionnaires compared to accelerometers is that the latter might have difficulties detecting upper body movement. As older people generally walk less but are still active in household activities, for this specific target population a self-reported questionnaire is the designated way of measurement [76]. Fourthly, apart from the aforementioned way of recruitment, there are some other differences between the intervention group and the reference group that have to be taken into account when comparing the two groups, such as the time of collecting information (2016 in Active Plus65 versus 2011 in Active Plus50) and the way of entry (free choice in Active Plus65 versus assigned in Active Plus50). Although the time lapse between 2016 and 2011 is a considerable amount of time, no research has been found that shows major shifts in PA behavior for older adults during this period. An increase in the use and availability of the internet among older adults in Europe can be seen in this time lapse [77], but this will most likely only have affected the preference for internet-based way of entry, which is accounted for as a covariate. Seasonal influences may have played a role but are difficult to determine due to differences in inclusion periods: the inclusion period of Active Plus50 lasted from November through to March, and that of Active Plus65 lasted from April through to May; as both interventions started in winter/spring, the difference in seasonal effects between both interventions can considered to be minor. A control group that did not undergo an intervention was not possible due to the implementation setting of Active Plus65 and the low number of suited participants in Active Plus50. However, study design that was employed allowed for a comparison of the adapted version of the intervention, i.e., Active Plus65, with its predecessor, and non-adapted version, Active Plus50, thus giving insights into the effects of the adaptations. Finally, when performing the a priori power analyses, Ess of the original Active Plus50 study [28] were used. In that study, an intervention group was compared to a no intervention control group, probably resulting in larger ESs than in the comparison of Active Plus65 with the reference group that, in contrast to the control group in Active Plus50, did receive an intervention. Post hoc analyses on achieved power showed that, for the found effect on the outcome measure of weekly minutes of MVPA between TO and T2, a power of 68% was achieved, which is relatively low, but acceptable.

## 5. Conclusions

Notwithstanding the abovementioned limitations, it can be concluded from the present findings that PA showed a significant increase in the Active Plus65 group, with a higher ES at three months than the average computer-tailored intervention, potentially making it an interesting healthy aging intervention to implement on a larger scale among the vulnerable target population of single older adults with a chronic impairment in PA. Active Plus65 did not outperform its predecessor Active Plus50, but the larger amount of participants with severe physical impairments in Active Plus65 may be accountable for this. In further development of Active Plus65, extra attention may be given to factors that support the long-term maintenance of the intervention effects, and to strategies that are particularly suitable for this target group with its specific characteristics.

## Figures and Tables

**Table 1:** Baseline characteristics of the research groups.

Variables	Active Plus65	Reference Group	*p*-Value
	(*n* = 416)	(*n* = 86)	
Days per week with sufficient MVPA (mean ± SD)	2.36 (2.31)	3.46 (2.24)	<0.001 *
Weekly minutes of MVPA (mean ± SD)	491.49 (±635.63)	539.28 (±616.18)	0.526
Age (years) (mean ± SD)	76.75 (±7.75)	74.36 (±6.26)	0.002 *
Gender (% male)	35.6%	22.4%	0.018 *
Education (% low)	67.4%	62.8%	0.413
BMI (mean ± SD)	27.3 (±5.05)	26.8 (±5.0)	0.390
Way of entry (% online)	41.1%	30.2%	0.060
Degree of impairment (% very to extremely impaired)	51.8%	32.6%	0.002 *
Intention to be physically active	6.712 (±1.67)	6.48 (±2.13)	0.355

* Significantly different between Active Plus65 and reference group. MVPA: Moderate to vigorous physical activity. Note: Degree of impairment is measured differently in Active Plus65 and the reference group, as explained in the Demographics section.

**Table 2:** Difference in physical activity (PA)-outcomes between Active Plus65 and reference group.

**Effects on Days per Week with Sufficient MVPA**	**B**	**SE**	***p***	**95% CI**	**ES**
T1	−0.18	0.36	0.626	−0.89/0.53	0.07
T2	0.29	0.40	0.473	−0.50/1.08	0.03
**Effects on Weekly Minutes of MVPA**	**B**	**SE**	***p***	**95% CI**	**ES**
T1	205.03	118.48	0.084	−27.88/437.93	0.11
T2	370.94	127.18	0.004	120.96/620.91	0.25

MVPA: moderate to vigorous physical activity; *Note*: Active Plus65 is coded as 0 and the reference group is coded as 1.

## Abbreviations

BMI	Body Mass Index
ES	Effect Size
MVPA	Minutes of moderate to vigorous physical activity
NCCD	Non-communicable chronic disease
PA	Physical activity
RCT	Randomized clinical trial

## References

[1] World Health Organisation (2015). World Report on Ageing and Health.

[2] Organisation for Economic Cooperation and Development Expenditure by Disease, Age and Gender. http://www.oecd.org/els/health-systems/Expenditure-by-disease-age-and-gender-FOCUS-April2016.pdf.

[3] European Union (2015). Quality of Life: Facts and Views.

[4] Ding D., Lawson K.D., Kolbe-Alexander T.L., Finkelstein E.A., Katzmarzyk P.T., van Mechelen W., Pratt M. (2016). The economic burden of physical inactivity: A global analysis of major non-communicable diseases. Lancet.

[5] McKenna M., Collins J., Remington P.L., Wegner M.V., Brownson R.C. (2010). Current issues and challenges in chronic disease control. Chronic Disease Epidemiology and Control.

[6] Goodman R.A., Posner S.F., Huang E.S., Parekh A.K., Koh H.K. (2013). Defining and measuring chronic conditions: Imperatives for research, policy, program, and practice. Prev. Chronic Dis..

[7] Matheson G., Klügl M., Engebretsen L., Bendiksen F., Blair S., Börjesson M., Budgett R., Derman W., Erdener U., Ioannidis J. (2013). Prevention and management of non-communicable disease: The ioc consensus statement, lausanne 2013. Sports Med..

[8] World Health Organisation Global Recommendations on Physical Activity for Health. http://www.who.int/dietphysicalactivity/publications/9789241599979/en/.

[9] Hamer M., Lavoie K.L., Bacon S.L. (2014). Taking up physical activity in later life and healthy ageing: The English longitudinal study of ageing. Br. J. Sports Med..

[10] Ekelund U., Steene-Johannessen J., Brown W.J., Fagerland M.W., Owen N., Powell K.E., Bauman A., Lee I.M. (2016). Does physical activity attenuate, or even eliminate, the detrimental association of sitting time with mortality? A harmonised meta-analysis of data from more than 1 million men and women. Lancet.

[11] Gajewski P., Falkenstein M. (2016). Physical activity and neurocognitive functioning in aging—A condensed updated review. Eur. Rev. Aging Phys. Act..

[12] World Health Organisation Global Report on Falls Prevention in Older Adults. https://books.google.nl/books?hl=nl&lr=&id=ms9o2dvfaQkC&oi=fnd&pg=PA1&dq=world+health+organisation+balance+older+adults&ots=5JFZHDULVP&sig=t88_W268r7Em4gkAayyDsBjZAMA#v=onepage&q=world%20health%20organisation%20balance%20older%20adults&f=false.

[13] Dutch Department of Health, Welfare and Sports Physical Activity Guidelines. http://www.webcitation.org/6rG9EZmy4.

[14] Sun R., Norman J., While A. (2013). Physical activity in older people: A systematic review. BMC Public Health.

[15] Newsom J.T., Huguet N., McCarthy M.J., Ramage-Morin P., Kaplan M.S., Bernier J., Bentson M., McFarland M., Oderkirk J. (2012). Health hehavior change following chronic illness in middle and later life. J. Gerontol. Psycholol. Soc. Sci..

[16] Öztürk A., Şimşek T.T., Yümin E.T., Sertel M., Yümin M. (2011). The relationship between physical, functional capacity and quality of life (qol) among elderly people with a chronic disease. Arch. Gerontol. Geriatr..

[17] World Health Organisation World Report on Disability. http://www.who.int/disabilities/world_report/2011/en/.

[18] Dutch Department of Health, Welfare and Sports Physical activity and Sports Behavior of People with a Chronic Condition. http://www.rivm.nl/Documenten_en_publicaties/Algemeen_Actueel/Nieuwsberichten/2015/Mensen_met_een_beperking_sporten_minder.

[19] World Health Organisation Prevalence of Insufficient Physical Activity. http://www.who.int/gho/ncd/risk_factors/physical_activity_text/en/.

[20] Cattan M., White M., Bond J., Learmouth A. (2005). Preventing social isolation and loneliness among older people: A systematic review of health promotion interventions. Ageing Soc..

[21] Hawkley L.C., Thisted R.A., Cacioppo J.T. (2009). Loneliness predicts reduced physical activity: Cross-sectional & longitudinal analyses. Health Psychol..

[22] Hawton A., Green C., Dickens A.P., Richards S.H., Taylor R.S., Edwards R., Greaves C.J., Campbell J.L. (2014). The impact of social isolation on the health status and health-related quality of life of older people. Qual. Life.

[23] Muellmann S., Forberger S., Mollers T., Broring E., Zeeb H., Pischke C.R. (2017). Effectiveness of ehealth interventions for the promotion of physical activity in older adults: A systematic review. Prev. Med..

[24] Elbert N.J., van Os-Medendorp H., van Renselaar W., Ekeland A.G., Hakkaart-van Roijen L., Raat H., Nijsten T.E.C., Pasmans S.G.M.A. (2014). Effectiveness and cost-effectiveness of ehealth interventions in somatic diseases: A systematic review of systematic reviews and meta-analyses. J. Med. Int. Res..

[25] World Health Organisation (2010). Global Status Report on Non-Communicable Diseases 2014.

[26] Boekhout J.M., Peels D.A., Berendsen B.A.J., Bolman C.A.W., Lechner L. (2017). An ehealth intervention to promote physical activity and social network of single, chronically impaired older adults: Adaptation of an existing intervention using intervention mapping. JMIR Res. Protoc..

[27] Peels D.A., Bolman C., Golsteijn R.H.J., De Vries H., Mudde A.N., Van Stralen M.M., Lechner L. (2013). Long-term efficacy of a tailored physical activity intervention among older adults. Int. J. Behavior Nutr. Phys. Act..

[28] Peels D.A., Van Stralen M.M., Bolman C., Golsteijn R.H.J., De Vries H., Mudde A.N., Lechner L. (2014). The differentiated (short-term) effectiveness of a printed versus a web-based tailored intervention to promote physical activity among the over-fifties: A randomized controlled trial. Health Educ. Res..

[29] Curran G.M., Bauer M., Mittman B., Pyne J.M., Stetler C. (2012). Effectiveness-implementation hybrid designs: Combining elements of clinical effectiveness and implementation research to enhance public health impact. Med. Care.

[30] Glasgow R.E., Vogt T.M., Boles S.M. (1999). Evaluating the public health impact of health promotion interventions: The re-aim framework. Am. J. Public Health.

[31] Bartholomew L.K., Markham C.M., Ruiter R.A.C., Fernandez M.E., Kok G., Parcel G.S. (2016). Planning Health Promotyion Programs: An Intervention Mapping Approach.

[32] De Vries H., Mudde A., Leijs I., Charlton A., Vartiainen E., Buijs G., Clemente M., Storm H., Navarro A., Nebot M. (2003). The European smoking prevention framework approach (EFSA): An example of integral prevention. Health Educ. Res..

[33] Prochaska J.O., Redding C.A., Evers K.E., Glanz K., Rimer B.K., Viswanath K. (2008). The transtheoretical model and stages of change. Health Behaviour and Health Education: Theory, Research, and Practice.

[34] Ryan R., Deci E.L. (2000). Self-determination theory and the facilitation of intrinsic motivation, social development, and well-being. Am. Psychol..

[35] Baumeister R.F., Vohs K.D. (2004). Handbook of Self-Regulation: Research, Theory, and Applications.

[36] Schwarzer R., Luszczynska A. (2008). How to overcome health-compromising behaviors: The health action process approach. Eur. Psychol..

[37] Peels D.A., Van Stralen M.M., Bolman C., Golsteijn R.H.J., De Vries H., Mudde A.N., Lechner L. (2012). The development of a web-based computer tailored advice to promote physical activity among people older than 50 years. J. Med. Int. Res..

[38] Van Stralen M.M., Kok G., De Vries H., Mudde A.N., Bolman C., Lechner L. (2008). The active plus protocol: Systematic development of two theory and evidence-based tailored physical activity interventions for the over-fifties. BMC Public Health.

[39] Hobbs N., Godfrey A., Lara J., Errington L., Meyer T.D., Rochester L., White M., Methers J.C., Sniehotta F.F. (2013). Are behavioural interventions effective in increasing physical activity at 12 to 36 months in adults aged 55 to 70 years? A systematic review and meta-analysis. BMC Med..

[40] Guertler D., Vandelanotte C., Kirwan M., Duncan M.J. (2015). Engagement and nonusage attrition with a free physical activity promotion program: The case of 10,000 steps Australia. J. Med. Int. Res..

[41] Neve M.J., Collins C.E., Morgan P.J. (2010). Dropout, nonusage attrition, and pretreatment predictors of non usage attrition in a commercial web-based weight loss program. J. Med. Int. Res..

[42] Wanner M., Martin-Diener E., Braun-Fahrländer C., Bauer G., Martin B.W. (2009). Effectiveness of active-online, an individually tailored physical activity intervention, in a real-life setting: Randomized controlled trial. J. Med. Int. Res..

[43] Faul F., Erdfelder E., Lang A.-G., Buchner A. (2007). G*power 3: A flexible statistical power analysis program for the social, behavioral, and biomedical sciences. Behav. Res. Methods.

[44] De Hollander E.L., Zwart L., De Vries S.I., Wendel-Vos W. (2012). The squash was a more valid tool than the obin for categorizing adults according to the dutich physical activity guideline. J. Clin. Epidemiol..

[45] Wagenmakers R., Van den Akker-Scheek I., Groothoff J.W., Zijlstra W., Bulstra S.K., Kootstra J.W.J., Wendel-Vos W., van Raaij J.J.A.M., Stevens M. (2008). Reliability and validity of the short questionnaire to assess health-enhancing physical activty (SQUASH) in patients after total hip arthroplasty. BMC Muskuloskeletal Disord..

[46] Wendel-Vos G.C., Schuit A.J., Saris W.H., Kromhout D. (2003). Reproducibility and relative validity of the short questionnaire to assess health-enhancing physical activity. J. Clin. Epidemiol..

[47] Fishbein M., Ajzen I. (2010). Predicting and Changing Behavior: The Reasoned Action Approach.

[48] Sheeran P., Orbell S. (1999). Implementation intentions and repeated behaviour: Augmenting the predictive validity of the theory of planned behaviour. Eur. J. Soc. Psychol..

[49] Van Stralen M.M., De Vries H., Bolman C., Mudde A.N., Lechner L. (2009). Determinants of initiation and maintenance of physical activity in older adults: A literature review. Health Psychol. Rev..

[50] Van Stralen M.M., de Vries H., Mudde A.N., Bolman C., Lechner L. (2011). The long-term efficacy of two computer-tailored physical activity interventions for older adults: Main effects and mediators. Health Psychol..

[51] Twisk J., de Boer M., de Vente W., Heymans M. (2013). Multiple imputation of missing values was not necessary before performing a longitudinal mixed-model analysis. J. Clin. Epidemiol..

[52] Morris S.B., DeShon R.P. (2002). Combining effect size estimates in meta-analysis with repeated measures and independent-groups designs. Psychol. Methods.

[53] York University, D.O.P. Effect Size Calculator. http://www.yorku.ca/ncepeda/effectsize.html.

[54] University of Colorado Effect Size Calculator. https://www.uccs.edu/~lbecker/.

[55] Cohen J. (1992). A power primer. Psychol. Bull..

[56] Gardner B., Smith L., Lorencatto F., Hamer M., Biddle S.J.H. (2016). How to reduce sitting time? A review of behaviour change strategies used in sedentary behaviour reduction interventions among adults. Health Psychol. Rev..

[57] Bankoski A., Harris T.B., McClain J.J., Brychta R.J., Caserotti P., Chen K.Y., Berrigan D., Troiano R.P., Koster A. (2011). Sedentary activity associated with metabolic syndrome independent of physical activity. Diabetes Care.

[58] Van der Ploeg H.P., Chey T., Korda R.J., Banks E., Bauman A. (2012). Sitting time and all-cause mortality risk in 222,497 Australian adults. Arch. Int. Med..

[59] Duvivier B., Schaper N., Hesselink M., Kan L., Stienen N., Winkens B., Koster A., Savelberg H. (2017). Breaking sitting with light activities vs. structured exercise: A randomised crossover study demonstrating benefits for glycaemic control and insulin sensitivity in type 2 diabetes. Diabetologia.

[60] Milton K., Clemes S., Bull F. (2013). Can a single question provide an accurate measure of physical activity?. Br. J. Med..

[61] Wanner M., Probst-Hensch N., Kriemler S., Meier F., Bauman A., Martin B.W. (2014). What physical activity surveillance needs: Validity of a single-item questionnaire. Br. J. Sports Med..

[62] Greaves C.J., Sheppard K.E., Abraham C., Hardeman W., Roden M., Evans P.H., Schwarz P. (2011). Systematic review of reviews of intervention components associated with increased effectiveness in dietary and physical activity interventions. BMC Public Health.

[63] Kwasnicka D., Dombrowski S.U., White M., Sniehotta F. (2016). Theoretical explanations for maintenance of behaviour change: A systematic review of behaviour theories. Health Psychol. Rev..

[64] Ory M.G., Smith M.L., Mier N., Wernicke M.M. (2010). The science of sustaining health behavior change: The health maintenance consortium. Am. J. Health Behav..

[65] Brouwer W.B.F., Kroeze W., Crutzen R., De Nooijer J., De Vries N., Brug J., Oenema A. (2011). Which intervention characteristics are related to more exposure to internet-delivered healthy lifestyle promotion interventions? A systematic review. J. Med. Int. Res..

[66] Greaney M.L., Sprunck-Harrild K., Bennett G.G., Puleo E., Haines J., Viswanath K.V., Emmons K.M. (2012). Use of email and telephone prompts to increase self-monitoring in a web-based intervention: Randomized controlled trial. J. Med. Int. Res..

[67] Schneider F., de Vries H., Candel M., van de Kar A., van Osch L. (2013). Periodic email prompts to re-use an internet-delivered computer-tailored lifestyle program: Influence of prompt content and timing. J. Med. Int. Res..

[68] Schneider F., van Osch L., Schulz D.N., Kremers S.P.J., de Vries H. (2012). The influence of user characteristics and a periodic email prompt on exposure to an internet-delivered computer-tailored lifestyle program. J. Med. Int. Res..

[69] Chase J.D. (2015). Interventions to increase physical activity among older adults: A meta-analysis. Gerontologist.

[70] Conn V.S., Hafdahl A.R., Brown S.A., Brown L.M. (2008). Meta-analysis of patient education interventions to increase physical activity among chronically ill adults. Patient Educ. Couns..

[71] Dishman R.K., Buckworth J. (1996). Increasing physical activity: A quantitative synthesis. Med. Sci. Sports Exerc..

[72] Eysenbach G. (2005). The law of attrition. J. Med. Int. Res..

[73] Adams K.B., Leibbrandt S., Moon H. (2011). A critical review of the literature on social and leisure activity and wellbeing in later life. Ageing Soc..

[74] Brown W.J., Burton N.W., Marshall A.L., Miller Y.D. (2008). Reliability and validity of a modified self-administered version of the active Australia physical activity survey in a sample of mid-age women. Aust. N. Z. J. Public Health.

[75] Webster S., Khan A., Nitz J.C. (2011). A brief questionnaire is able to measure population physical activity levels accurately: A comparative validation study. J. Clin. Gerontol. Geriatr..

[76] Harris T.J., Owen C.G., Victor C.R., Adams R., Ekelund U., Cook D.G. (2009). A comparison of questionnaire, accelerometer, and pedometer: Measures in older people. Med. Sci. Sports Exerc..

[77] European Union Digital Economy and Society Statistics: Households and Individuals. http://ec.europa.eu/eurostat/statistics-explained/index.php/Digital_economy_and_society_statistics_-_households_and_individuals.

